# FMRP Enhances the Translation of *4EBP2* mRNA during Neuronal Differentiation

**DOI:** 10.3390/ijms242216319

**Published:** 2023-11-14

**Authors:** Jinbae Yu, Youngsik Woo, Heesun Kim, Sihyeon An, Sang Ki Park, Sung Key Jang

**Affiliations:** Department of Life Sciences, Pohang University of Science and Technology (POSTECH), Pohang 37673, Gyeongsangbuk, Republic of Korea; jhjqlsujb@postech.ac.kr (J.Y.); youngsik.woo@postech.ac.kr (Y.W.); heesun@postech.ac.kr (H.K.); there1@postech.ac.kr (S.A.)

**Keywords:** FMRP, 4EBP2, translation enhancement, neurite outgrowth, FXS

## Abstract

FMRP is a multifunctional protein encoded by the *Fragile X Messenger Ribonucleoprotein 1* gene (*FMR1*). The inactivation of the *FMR1* gene results in fragile X syndrome (FXS), a serious neurodevelopmental disorder. FMRP deficiency causes abnormal neurite outgrowth, which is likely to lead to abnormal learning and memory capabilities. However, the mechanism of FMRP in modulating neuronal development remains unknown. We found that FMRP enhances the translation of 4EBP2, a neuron-specific form of 4EBPs that inactivates eIF4E by inhibiting the interaction between eIF4E and eIF4G. Depletion of 4EBP2 results in abnormal neurite outgrowth. Moreover, the impairment of neurite outgrowth upon FMRP depletion was overcome by the ectopic expression of 4EBP2. These results suggest that FMRP controls neuronal development by enhancing 4EBP2 expression at the translational level. In addition, treatment with 4EGI-1, a chemical that blocks eIF4E activity, restored neurite length in FMRP-depleted and 4EBP2-depleted cells. In conclusion, we discovered that 4EBP2 functions as a key downstream regulator of FMRP activity in neuronal development and that FMRP represses eIF4E activity by enhancing 4EBP2 translation.

## 1. Introduction

FXS, which results from the inactivation of the *FMR1* gene encoding FMRP, is a representative example of monogenic neurological disorders [1,2,3,4]. The prevalence of FXS is estimated to be 1 in 4000 in males and 1 in 8000 in females [5]. The characteristic phenotype of individuals with FXS is distinguished by distinctive craniofacial features, including prominent ears and chin, as well as a long and narrow facial structure [6]. The main symptoms of FXS include attention deficit, hyperactivity, autism-like behavior, impulsivity, and seizures [7]. Despite a comprehensive understanding of FXS, the absence of approved therapeutic interventions for patients with FXS is still evident. Therefore, it is necessary to improve our understanding of the intricate molecular mechanisms through which the dysregulation of *FMR1* and FMRP leads to FXS. 

FMRP is an RNA-binding protein that regulates more than 800 mRNAs, including those related to synaptic functions and autism spectrum disorder (ASD) [8]. However, the FMRP-mediated control of downstream gene expression is difficult to discern because it is a multifunctional protein regulating the translation, localization, and stabilization of target RNAs [1] via three distinct RNA-binding domains [9]. FMRP is known to function as a translational repressor, either by preventing the formation of the eIF4F complex at the initiation step [10] or by stalling polyribosomes at the elongation step [8,11]. However, it has recently been suggested to enhance the translation of some mRNAs [12]. For instance, *dFMR1*, the FMRP homologue in *Drosophila*, enhances the translation of mRNAs associated with intellectual disability and autism in *Drosophila* oocytes [13]. The expression of certain proteins such as Sod1 [14], Kv4.2 [15], and Dgkκ [16] was reduced in *FMR1* null mice, and transfection of siRNAs showed a positive correlation between FMRP and hASH1 in the human brain [17]. Moreover, human NOS1 translation was activated by FMRP in the developing neocortex [18]. In spite of these examples, the molecular mechanism of FMRP-mediated translational activation remains to be elucidated.

eIF4E plays an important role in translation by recruiting translation factors to the 5′-end of mRNA through direct interactions with the 7-methyl guanosine cap structure [19,20]. Although eIF4E is considered a general translation factor participating in the translation of almost all capped mRNAs, recent eIF4E modulation experiments have suggested that eIF4E regulates the translation of selective groups of mRNAs with highly structured sequences in the 5′ untranslated region (5′UTR) [21] or specific sequence elements [22,23].

The loss of normal constraints on protein synthesis may be one of the pathogenic mechanisms leading to synaptic and cognitive impairment caused by an FMRP defect [24]. Moreover, the reduced expression of FMRP has been reported to be associated with the hyperactivation of eIF4E through the phosphorylation of eIF4E [25]. In addition, a compound named 4EGI-1, which inhibits eIF4E activity by blocking eIF4F complex formation by directly binding to eIF4E [26], improves the hippocampal synaptic function, dendritic morphology, and learning behaviors in FXS mice models [27]. In fact, ASD-like behaviors and altered synaptic functions, similar to the symptoms observed in FXS model mice [28], were generated in mice, either by the overexpression of eIF4E [29] or by genetic deletion of eIF4E-binding protein 2 (4EBP2) [30], which is the major 4EBP species produced in neuronal cells [31] that inactivates eIF4E function by blocking eIF4F complex formation [20,32,33]. Moreover, the ASD-like behavior of mice caused by 4EBP2 knockout was neutralized by 4EGI-1 treatment [30]. These reports indicate that properly controlled eIF4E activity is required for the normal function of neuronal cells, and that a proper balance between eIF4E and its antagonistic protein 4EBP2 appears to be necessary to avoid neurological disorders such as FXS. However, our understanding of the relationship between FMRP and 4EBP remains limited. 

In the early stages of neuronal development, cells project a number of neurites from the cell body in a process called neurite outgrowth, and these become axons and dendrites at later stages [34,35]. Neurite development is controlled by a complex combination of genes encoding cytoskeletal regulators, surface receptors, adhesion molecules, synaptic proteins, and signaling molecules [36]. Incomplete neurite outgrowth mediated by abnormal gene expression is often followed by defects in synaptic functionality [37], which contribute to learning and memory deficits [38]. The induced pluripotent stem cells (iPSCs) [39], human embryonic stem cells (hESCs) [37,40,41], and differentiated neural stem cells (NSCs) [42] derived from patients with FXS exhibit shortened neurite lengths, leading to improper signal transduction, similar to FXS pathology. However, the molecular basis for this defect in neurite outgrowth in FMRP-deficient cells remains unclear.

Here, we report that FMRP depletion reduces 4EBP2 expression in human SH-SY5Y neuroblastoma cells under neuronal differentiation conditions, indicating that FMRP is required for the enhanced translation of *4EBP2* mRNA during neuronal development. Moreover, we show that the 5′UTR of *4EBP2* mRNA, where FMRP binds, is responsible for the FMRP-mediated translational enhancement of *4EBP2* mRNA. We additionally observe that FMRP depletion from mouse primary cultured hippocampal neurons by shRNAs against *FMR1* mRNA resulted in reduced neurite outgrowth. Reduced neurite outgrowth from mouse neuronal cells upon FMRP depletion was overcome by the ectopic expression of 4EBP2 or by the treatment of 4EGI-1. These results strongly suggest that FMRP plays a critical role in promoting optimal neurite outgrowth via the translational enhancement of *4EBP2* mRNA.

## 2. Results

### 2.1. FMRP Enhances the Expression of 4EBP2 in Neuronal Cells

FMRP depletion results in hyperactivation of the eIF4E-dependent translation pathway [25]. 4EBP2, a neuron-specific homologue of 4EBP1, represses eIF4E in neuronal cells by binding to eIF4E [20,31,32,33]. Moreover, 4EBP2, similar to FMRP [8,24,27], is known to participate in the regulation of synaptic function in brain tissue [31], and a defect in 4EBP2 results in a phenotype similar to that in ASD [30]. Based on these reports, we hypothesized that the pathological disorders in FXS, which result from FMRP defects, might be attributed to putative abnormal expression of 4EBP2 caused by the FMRP defects.

**Figure 1:**
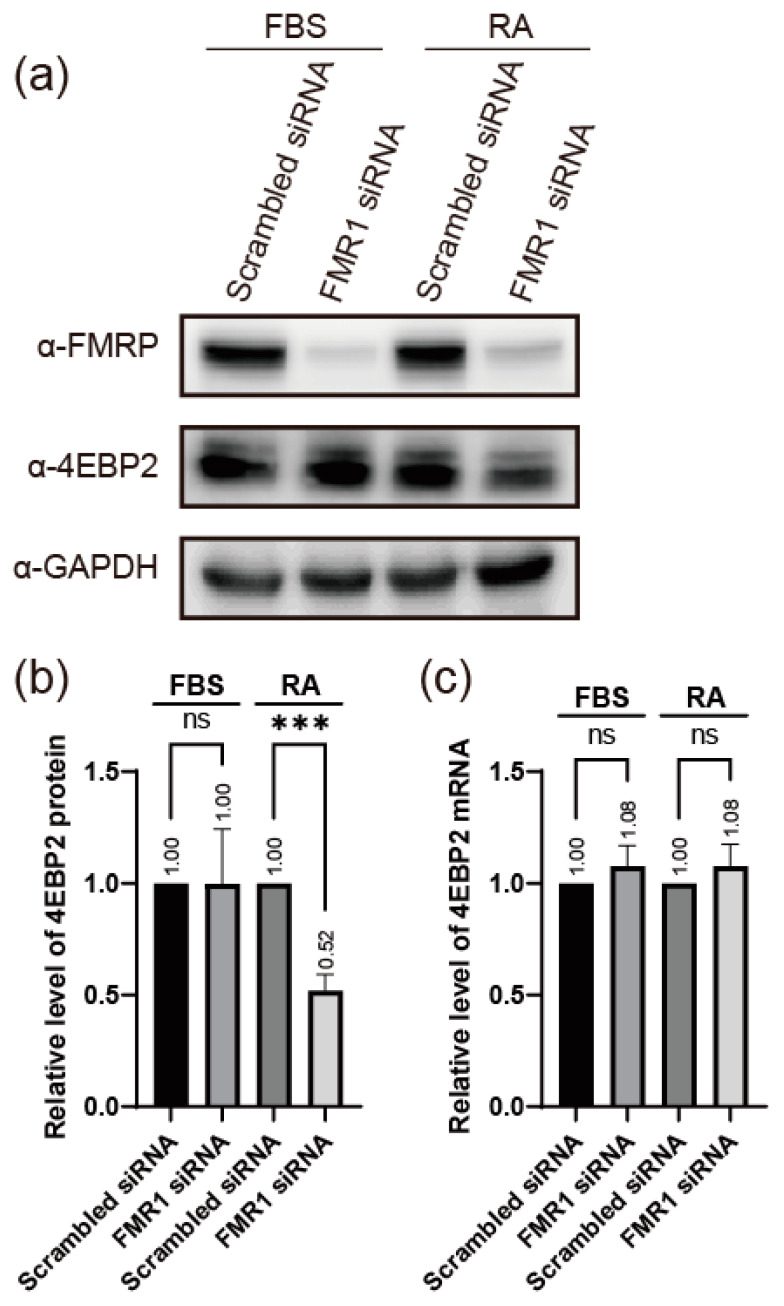
FMRP enhances 4EBP2 expression. (**a**) The amounts of proteins (FMRP, 4EBP2, and GAPDH) in SH-SY5Y cells under neuron induction conditions were monitored via Western blot analysis using the specified antibodies. Indicated siRNAs were transfected into cells after 24 h of cell culture. The culture medium was replaced with fresh medium containing 10% (*v*/*v*) FBS or 10 μM RA 24 h after siRNA transfection. Cells were lysed 48 h after the media change. (**b**) Relative amounts of 4EBP2 protein shown in panel (**a**) are depicted in the graph. The level of 4EBP2 was normalized to that of GAPDH, and the level of 4EBP2 in the cells treated with Scrambled siRNA was set to ‘1’ for each condition. (**c**) The relative amounts of *4EBP2* mRNA in SH-SY5Y cells treated as described in panel (**a**), were measured using quantitative RT-PCR and are depicted in the graph. Three or more independent repetitions were performed for each experiment. All data are represented as means ± SD. In one-way ANOVA, the asterisk *** presents *p* < 0.001. *p* values over 0.05 are marked as non-significant (ns).

We tested this hypothesis by observing the levels of 4EBP2 in the FMRP knockdown cells. We used the human neuroblastoma cell line SH-SY5Y, which can be differentiated into neuron-like cells by treatment with retinoic acid (RA) [43,44]. In undifferentiated cells, knockdown of *FMR1* mRNA did not result in any change in the expression of 4EBP2 (lane FBS in Figure 1a,b). In contrast, the expression of 4EBP2 was substantially reduced when FMRP levels were reduced by the knockdown of *FMR1* mRNA in neuron-like SH-SY5Y cells differentiated by RA treatment (lane RA in Figure 1a,b). The level of *4EBP2* mRNA was not affected by the depletion of FMRP, with or without differentiation of SH-SY5Y cells (Figure 1c). These results suggest that the expression of 4EBP2 is affected by FMRP at the post-transcriptional level in neuronal cells. Differentiation of SH-SY5Y cells by RA treatment was confirmed by monitoring the levels of neuronal marker mRNAs [43] 2 days after RA treatment (Supplementary Figure S1).

### 2.2. FMRP Enhances Translation of 4EBP2

**Figure 2:**
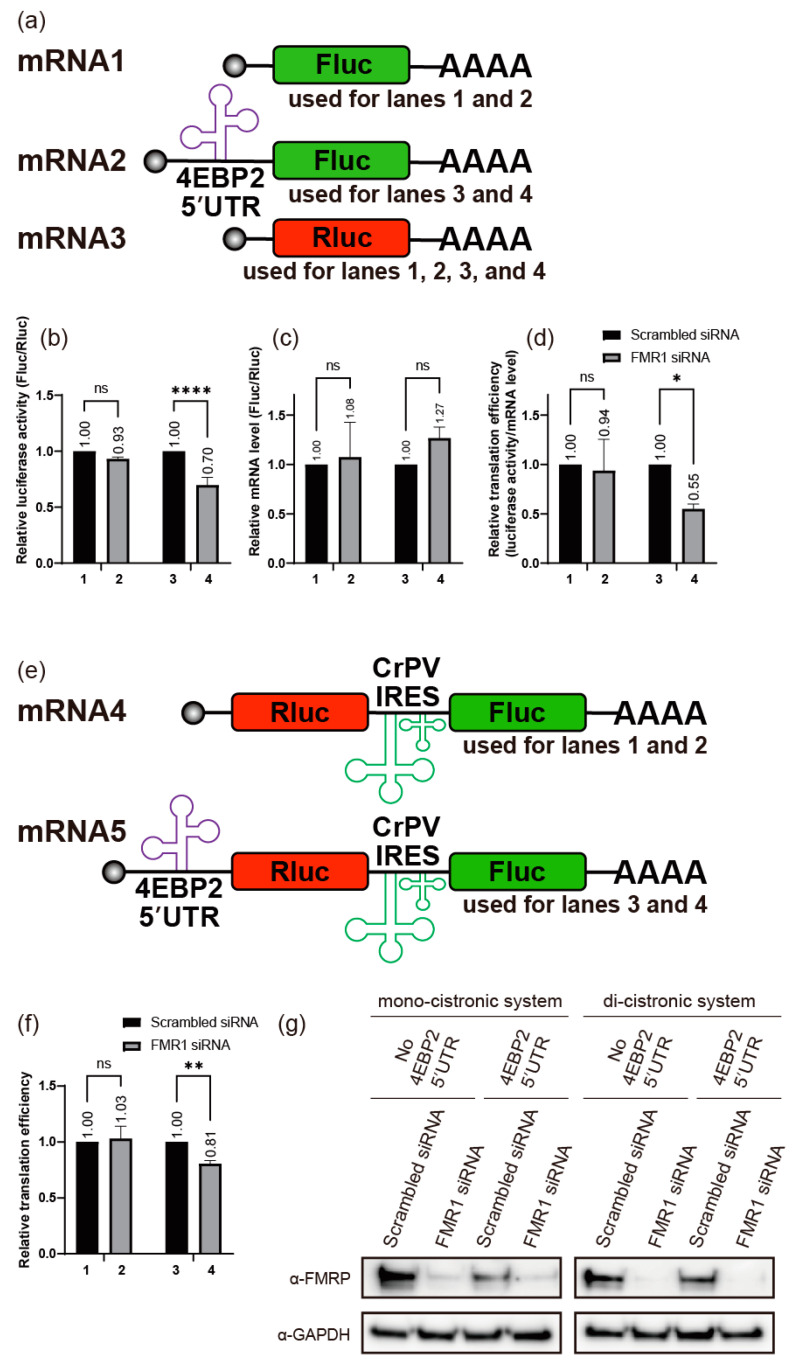
The *cis*-acting element required for FMRP-dependent translation resides in the 5′UTR of *4EBP2* mRNA. (**a**) Schematic diagram of the mono-cistronic mRNAs. All mRNAs contain the 5′ cap structure and 3′ poly(A) tail. The test mRNA (mRNA2) contains the 5′UTR of *4EBP2* mRNA followed by the *firefly* luciferase (Fluc) gene, but the negative control mRNA (mRNA1) contains about 60 nucleotides of sequence originating from the vector DNA at the 5′UTR followed by the Fluc gene. The reference mRNA (mRNA3), used for observing transfection efficiency and cell conditions, contains the *Renilla* luciferase (Rluc) gene. (**b**–**d**) SH-SY5Y cells were transfected with siRNAs (Scrambled siRNA for lanes 1 and 3 in panels (**b**–**d**); siRNA against *FMR1* mRNA for lanes 2 and 4 in panels (**b**–**d**)) after 24 h of cell culture. Plasmids expressing Fluc and Rluc mRNAs (mRNAs1 + 3 for lanes 1 and 2 in panels (**b**–**d**), or mRNAs2 + 3 for lanes 3 and 4 in panels (**b**–**d**)) were co-transfected into cells 24 h after siRNA transfection. The culture medium was replaced with a fresh medium containing 10 μM of RA 4 h after the DNA transfection. Following the medium change, cells were lysed, and both luciferase activity and the amount of mRNA in each lysate were measured after 48 h. (**b**) The relative luciferase activities (Fluc/Rluc values) under various conditions are depicted in panel (**b**), with the Fluc/Rluc value of cell lysate treated with Scrambled siRNA set to ‘1’, as shown by the columns on lanes 1 and 3. (**c**) The amounts of Fluc and Rluc mRNAs were measured by quantitative RT-PCR. The relative mRNA levels (Fluc mRNA/Rluc mRNA values) under various conditions are depicted in panel (**c**), with the Fluc/Rluc value of cell lysate treated by Scrambled siRNA set to ‘1’, as shown by the columns on lanes 1 and 3. (**d**) Translational efficiencies (T.E.) of reporter mRNAs were calculated by normalizing luciferase activities to RNA levels in each lysate. The average values of relative T.E. were calculated by the following formula [47,48,49]: ∑(Fluc T.E. ÷ Rluc T.E) ÷ number of experiments = ∑[(Fluc activity/Fluc mRNA level) ÷ (Rluc activity/Rluc mRNA level)] ÷ number of experiments. The average values of relative T.E. are depicted in panel (**d**), with the relative T.E. value of cell lysate treated by Scrambled siRNA set to ‘1’, as shown by the columns on lanes 1 and 3. (**e**) Schematic diagram of the di-cistronic mRNAs. All mRNAs contain the 5′cap structure and 3′ poly(A) tail. The test mRNA (mRNA5) contains the 5′UTR of *4EBP2* mRNA followed by the Rluc gene-CrPV IRES-Fluc gene, consecutively. Meanwhile, the negative control mRNA (mRNA4) contains about 60 nucleotides of a sequence originating from the vector DNA at the 5′UTR of the first cistron, followed by the Rluc gene-CrPV IRES-Fluc gene. The translation of Fluc, as directed by CrPV IRES, functions as a reference for the transfection efficiency of the plasmid. (**f**) Transfection of SH-SY5Y cells with siRNAs and plasmids and the analyses of reporter genes were performed as described in the legends to panels (**b**–**d**) except that plasmids expressing di-cistronic mRNAs (mRNA4 and mRNA5) were transfected into cells. Moreover, changes in Rluc activity relative to Fluc activity were analyzed as the Rluc gene was under the translational control of *4EBP2* 5′UTR in the di-cistronic mRNA. Therefore, the average values of relative T.E. were calculated by the following formula [50,51]: ∑(Rluc activity/Fluc activity) ÷ number of lysates. The average values of relative T.E. are depicted in panel (**f**), with the relative T.E. value of cell lysate treated by Scrambled siRNA set to ‘1’, as shown by the columns on lanes 1 and 3. (**g**) The knockdown efficiencies of siRNAs were monitored via Western blot analysis using the specified antibodies. Three or more independent repetitions were performed for each experiment. All data are represented as means ± SD. In two-way ANOVA, the asterisks *, **, and **** indicate *p* < 0.05, *p* < 0.01, and *p* < 0.0001, respectively. *p* values over 0.05 are marked as non-significant (ns).

As described above, FMRP enhances 4EBP2 expression at the post-transcriptional level in neuronal cells. We sought to understand the molecular basis of FMRP-mediated expression of 4EBP2 (Figure 1). We investigated the effects of the 5′UTR and the 3′UTR of *4EBP2* mRNA on the expression of a reporter gene, *firefly* luciferase (Fluc) gene, when the 5′UTR or the 3′UTR of *4EBP2* mRNA was artificially placed upstream or downstream of the reporter gene, respectively. We used the reporter *Renilla* luciferase (Rluc) gene as a reference mRNA in a mono-cistronic context (Figure 2a). In the case of the mono-cistronic mRNA format, each plasmid expressing reporter mRNA composed of a test mRNA (mRNA2) or a control mRNA (mRNA1, containing the Fluc gene but lacking the 5′UTR of *4EBP2* mRNA) was co-transfected together with a plasmid expressing the reference mRNA (mRNA3, containing the Rluc gene that reflects transfection efficiency) (Figure 2a–d). Translation of the Fluc gene containing the 5′UTR of *4EBP2* mRNA (mRNA2) was greatly reduced by treatment with an siRNA targeting *FMR1* mRNA (Figure 2d) in SH-SY5Y cells under neuronal differentiation conditions (compare lane 4 with 3 in Figure 2d). Fluc activity (Figure 2b) was normalized to the mRNA levels (Figure 2c) to calculate the relative translational efficiencies (T.E.) (Figure 2d). In contrast, the expression of the Fluc gene containing the 3′UTR of *4EBP2* mRNA was not affected by treatment with a siRNA targeting *FMR1* mRNA under neuronal differentiation conditions (Supplementary Figure S2e). Moreover, the expression of the reporter gene Fluc, containing either the 5′UTR or 3′UTR of *4EBP2* mRNA, was not affected by the treatment with siRNAs targeting *FMR1* mRNA, when SH-SY5Y cells were cultivated under normal conditions without RA treatment (Supplementary Figure S2f). These results indicate that FMRP enhances the expression of an mRNA containing the 5′UTR of *4EBP2* mRNA under neuronal differentiation conditions. We conclude that the regulation of 4EBP2 expression by FMRP occurs at the translational level via the 5′UTR of *4EBP2* mRNA because we used the same reporter genes, Fluc and Rluc, in measuring gene expression to minimize the effects of post-translational regulation of FMRP such as stability and subcellular localization, etc. (Figure 2b). We confirmed this hypothesis using di-cistronic mRNAs containing the *4EBP2* 5′UTR-Rluc-cricket paralysis virus (CrPV) internal ribosome entry site (IRES)-Fluc (mRNA5 in Figure 2e). CrPV IRES was used for the translation of the control reporter Fluc because CrPV IRES-mediated translation does not require any translation initiation factor [45,46]. In this di-cistronic mRNA format, the activities of the reference reporter Fluc reflect the mRNA levels and transfection efficiencies. The expression of Rluc under the control of 5′UTR of *4EBP2* mRNA was considerably reduced by the treatment with an siRNA targeting *FMR1* mRNA (Figure 2g) under neuronal differentiation conditions (compare lane 4 with 3 in Figure 2f). The absolute luciferase values in both mono-cistronic and di-cistronic systems (Supplementary Figure S2a–d) suggest that the observed effect is not due to an unintended increase in the normalizers (Rluc in the mono-cistronic system and Fluc in the di-cistronic system) following siRNA treatment. The findings obtained from the di-cistronic mRNA experiments decisively eliminate the possibility of FMRP’s involvement in regulating the expression of 4EBP2 through potential alterations in the stability or subcellular positioning of *4EBP2* mRNA. This is because both reporter genes are co-localized within a single mRNA molecule. Therefore, these results also strongly indicate that FMRP enhances translation of *4EBP2* mRNA via the 5′UTR of *4EBP2* mRNA during the differentiation of a neuronal cell.

### 2.3. FMRP Binds to the 5′UTR of 4EBP2 mRNA

**Figure 3:**
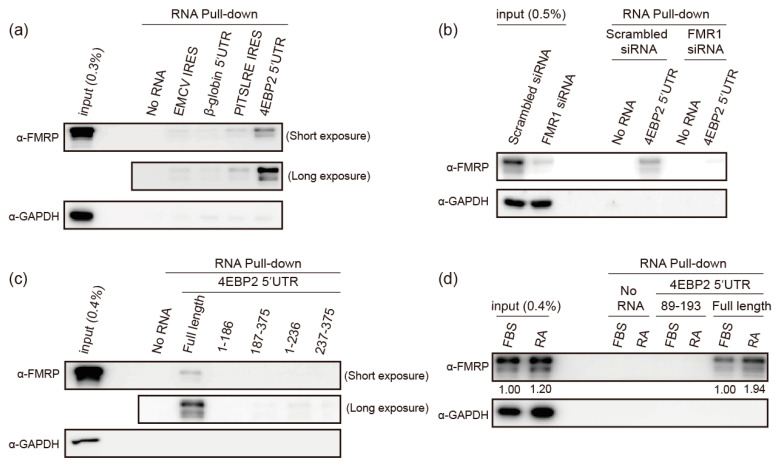
FMRP binds to the 5′UTR of *4EBP2* mRNA. (**a**–**d**) The lysates of SH-SY5Y cells and the indicated biotinylated RNAs were used in RNA pull-down assays. Streptavidin-conjugated agarose resins were used to precipitate RNA-bound proteins, which were then detected via Western blot analysis using the specified antibodies. (**a**) RNAs matching to EMCV IRES, β-globin 5′UTR, PITSLRE IRES, and *4EBP2* 5′UTR were used in the RNA pull-down assay. Western blotting using an anti-FMRP antibody was used to detect the FMRPs associated with the RNAs. Two different exposure times were used to clearly distinguish the RNA-binding affinities. GAPDH protein was used as a negative control. (**b**) RNA pull-down experiments were performed with or without FMRP knockdown to confirm that the band visualized via Western blotting represents FMRP. (**c**) Determination of FMRP binding site in the *4EBP2* 5′UTR. The regions in the *4EBP2* 5′UTR used in the RNA pull-down experiments are depicted by numbers. (**d**) The effect of neuronal differentiation on the binding of FMRP to the *4EBP2* 5′UTR. Two RNAs corresponding to the full-length *4EBP2* 5′UTR (full-length) and putative G-quadruplex-containing region of *4EBP2* 5′UTR (89–193) were used in RNA pull-down experiments using two different lysates of SH-SY5Ycells with (RA) or without (FBS) neuronal differentiation. Three or more independent repetitions were performed for each experiment.

We then aimed to investigate the mechanisms underlying FMRP-mediated modulation of the translation of *4EBP2* mRNA. Hence, we tested whether FMRP specifically interacts with the *4EBP2* 5′UTR using RNA pull-down assays. For this, we used biotinylated RNAs and lysates of SH-SY5Y cells with or without RA treatment because FMRP is an RNA-binding protein [9]. Among the RNAs, FMRP specifically bound to the full-length 5′UTR of *4EBP2* mRNA (Figure 3a and Figure S3). *FMR1* mRNA knockdown demonstrated that the band in Figure 3a corresponded to FMRP (Figure 3b). In contrast, truncated versions of the *4EBP2* 5′UTR (1–186, 187–375, 1–236, and 237–375) did not bind to FMRP (Figure 3c). These results indicate that a large part of *4EBP2* 5′UTR is required for the interaction with FMRP. Moreover, FMRP showed a stronger binding affinity to the full-length *4EBP2* 5′UTR under the neuronal differentiation conditions than that seen under undifferentiated conditions (Figure 3d). However, no significant binding of FMRP was observed to the region where the putative G-quadruplex (GQ) [52] structures, known as FMRP-binding motifs [53], were predicted to exist (89–193 in Figure 3d). The increased binding of FMRP to *4EBP2* 5′UTR, under neuronal differentiation conditions, might be related to the enhanced translation of 4EBP2 during neuronal differentiation.

### 2.4. DAP5 Enhances Translation of 4EBP2

Given the intricate nature of the multiple factors involved in translation initiation, conducting a comprehensive investigation of all mechanistic details of translation activation by *4EBP2* mRNA-bound FMRP within a single experiment is challenging. Nonetheless, we performed preliminary experiments to determine the mechanism of translational enhancement by FMRP. For this purpose, we sought to find a translation factor that potentially recruits the 40S ribosome to FMRP-bound mRNA. 

The translation initiation factor eIF4G1 (eIF4GI) and its homologues, such as eIF4G2 (death-associated protein 5: DAP5) and eIF4G3 (also known as eIF4GII), play key roles in translation for the recruitment of the 40S ribosomal subunit to mRNAs [54]. eIF4G1 and its homologues are scaffold proteins that are indirectly associated with the 40S subunit through interactions with eIF3 [54,55,56,57]. Among the eIF4G homologues, DAP5 has been suggested to participate in the regulation of embryonic development [58] and axonal outgrowth of hippocampal neurons [59], according to neurophysiological studies. Moreover, several studies have demonstrated physical interactions between FMRP and DAP5 [56,60]. However, the role of the interaction between DAP5 and FMRP during translation has not yet been investigated.

**Figure 4:**
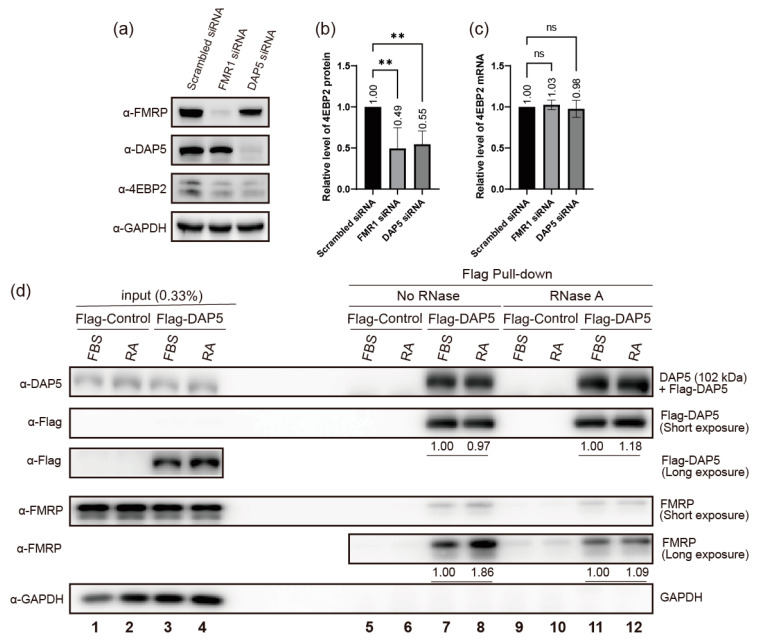
(**a**–**c**) The effects of FMRP- and DAP5-depletion on the expression of 4EBP2. (**a**) The amounts of proteins (FMRP, DAP5, 4EBP2, and GAPDH) in SH-SY5Y cells under neuron differentiation conditions were monitored via Western blot analysis using the specified antibodies. Indicated siRNAs were transfected into cells after 24 h of cell culture. The culture medium was replaced with fresh medium containing 10 μM retinoic acid (RA), 24 h after siRNA transfection. Cells were lysed 48 h after the media change. (**b**) Relative amounts of 4EBP2 protein shown in panel (**a**) are depicted in the graph. The level of 4EBP2 was normalized to that of GAPDH, and the 4EBP2 level in the cells treated with Scrambled siRNA was set to ‘1’ for comparison. (**c**) The relative amounts of *4EBP2* mRNA in SH-SY5Y cells (treated as described in panel (**a**)) were measured using qRT-PCR and are depicted in the graph. Three or more independent repetitions were performed for each experiment. All data are presented as means ± SD. In one-way ANOVA, the asterisk ** represents *p* < 0.01. *p* values over 0.05 are marked as non-significant (ns). (**d**) DAP5 interacts with FMRP in RNA-dependent and RNA-independent manners. The physical interaction between endogenous FMRP and Flag-tagged DAP5 (Flag-DAP5), which was ectopically expressed, was investigated by coimmunoprecipitation with a resin conjugated with a Flag-antibody. Flag-immunoprecipitation was performed with the lysates of SH-SY5Y cells expressing Flag-DAP5 with (RA, even number lanes) or without (FBS, odd number lanes) neuronal differentiation. The cell lysates were prepared with (lanes 9–12) or without (lanes 5–8) pre-treatment of RNase A to test the effect of RNAs on the FMRP-DAP5 interaction. The amounts of proteins (DAP5, Flag-DAP5, FMRP, and GAPDH) in the cell lysates before (lanes 1–4) and after (lanes 5–12) Flag pull-down were monitored via Western blot analysis using the specified antibodies. FMRP was coprecipitated with Flag-DAP5, even in the absence of RNAs (lanes 11 and 12 in panel α-FMRP). This indicates that FMRP-DAP5 interaction occurs in an RNA-independent manner. The FMRP-DAP5 interaction was not strengthened by neuronal differentiation in the absence of RNAs (compare lane 12 with 11 in panel α-FMRP). However, interestingly, the FMRP-DAP5 interaction was strongly increased by neuronal differentiation in the presence of RNAs (compare lane 8 with 7 in panel α-FMRP). This indicates that RNAs are required for the enhanced FMRP-DAP5 interaction during neuronal differentiation. Three or more independent repetitions were performed for each experiment.

To determine whether DAP5 regulates 4EBP2 expression during neuronal differentiation, we investigated the effect of DAP5 knockdown on the expression of 4EBP2 (Figure 4a–c). Our results showed that the knockdown of *DAP5* mRNA reduced the expression of 4EBP2, similar to the knockdown of *FMR1* mRNA (Figure 4b). We also investigated the interaction between FMRP and DAP5 by coprecipitation of proteins after ectopic expression of Flag-tagged DAP5 (Flag-DAP5). A similar amount of endogenous FMRP coprecipitated with Flag-DAP5 when RNase was added to the cell lysates with or without the differentiation of neuronal cells. (See lanes loaded with RNase-treated samples (RNase A) in Figure 4d) This indicates that protein–protein interactions between FMRP and DAP5 occur even in the absence of RNA. In contrast, a larger amount of FMRP coprecipitated with Flag-DAP5 during the differentiation of neuronal cells in the presence of RNAs. (See the lanes loaded with RNase-untreated samples (No RNase) in Figure 4d) This indicates that the amount of FMRP-DAP5 complex increases in an RNA-dependent manner during neuronal differentiation.

### 2.5. Depletion of FMRP Induces Defects in Neurite Outgrowth

**Figure 5:**
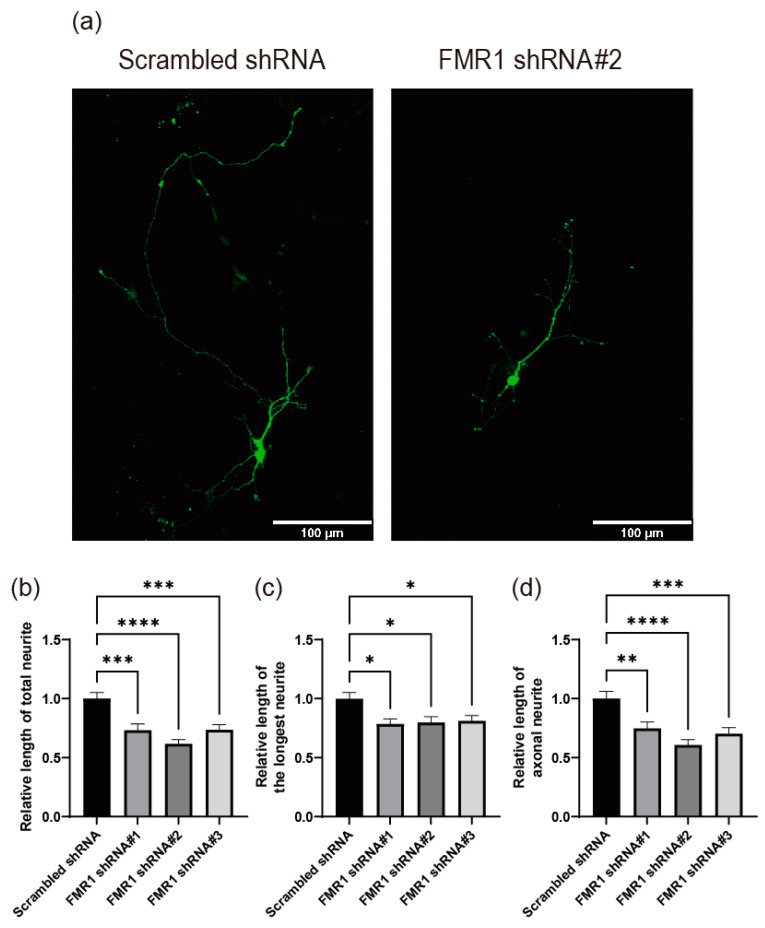
FMRP is involved in the regulation of neurite outgrowth of primary cultured hippocampal neurons. (**a**–**d**) Depletion of FMRP by shRNAs against *FMR1* mRNA impaired neurite outgrowth in primary cultured hippocampal neurons. Plasmids expressing shRNAs and a green fluorescent protein (GFP) encoding gene, which was used in the selection of shRNA-producing cells showing green fluorescence, were transfected into cells after 24 h of neuronal cell culture, and the morphologies of neurites extending from the neuronal cells were analyzed 4 days after in vitro cultivation of hippocampal neurons (DIV 4) (Scrambled shRNA, *n* = 43 cells; *FMR1* shRNA#1, *n* = 33 cells; *FMR1* shRNA#2, *n* = 41 cells; *FMR1* shRNA#3, *n* = 36 cells). (**a**) Representative pictures of the DNA-transfected neurons. The morphologies of neuronal cells expressing shRNAs (green) were analyzed and are depicted in the graphs (panels (**b**–**d**)). Utilizing the Simple neurite tracer plug-in of the ImageJ (version 1.52p) program, the relative lengths of the total neurite (**b**), longest neurite (**c**), and axonal neurite (**d**) were calculated. Each value was normalized to the average neurite length of cells expressing negative control (Scrambled) shRNA. Three shRNAs (#1, #2, and #3) with different core sequences were used. The scale bars in panel (**a**) represent 100 μm. Three or more independent repetitions were performed for each experiment. All data are presented as means ± SEM. In one-way ANOVA, the asterisks *, **, ***, and **** represent *p* < 0.05, *p* < 0.01, *p* < 0.001, and *p* < 0.0001, respectively.

Cells deficient in FMRP display atypical morphological characteristics, including shorter length of neurites, which suggests that FMRP may play a role in regulating proper neurite outgrowth [37,39,40,41,42]. However, the critical downstream proteins regulated by FMRP for normal neurite development remain unclear. To develop a cell-based system for the investigation of the key molecules functioning downstream of FMRP in neurite outgrowth, we used mouse primary cultured hippocampal neurons and an shRNA-mediated knockdown technique. We generated three shRNAs targeting *FMR1* mRNAs and tested their effects in NIH/3T3 cells derived from mice. Transfection with shRNA against *FMR1* mRNA efficiently reduced FMRP expression in NIH/3T3 cells (Supplementary Figure S4). Primary cultured mouse hippocampal neurons were used as model neurons in the presence of *FMR1* shRNA or Scrambled negative control shRNA (Figure 5). Under FMRP knockdown conditions, cultured hippocampal neurons exhibited substantially decreased neurite lengths (total, longest, and axonal) compared with control cells (Figure 5). These results suggest that the knockdown of *FMR1* in primary cultured mouse hippocampal neurons is a suitable system for investigating FMRP function.

### 2.6. 4EBP2 Is Epistatic to FMRP in the Modulation of Neurite Outgrowth

**Figure 6:**
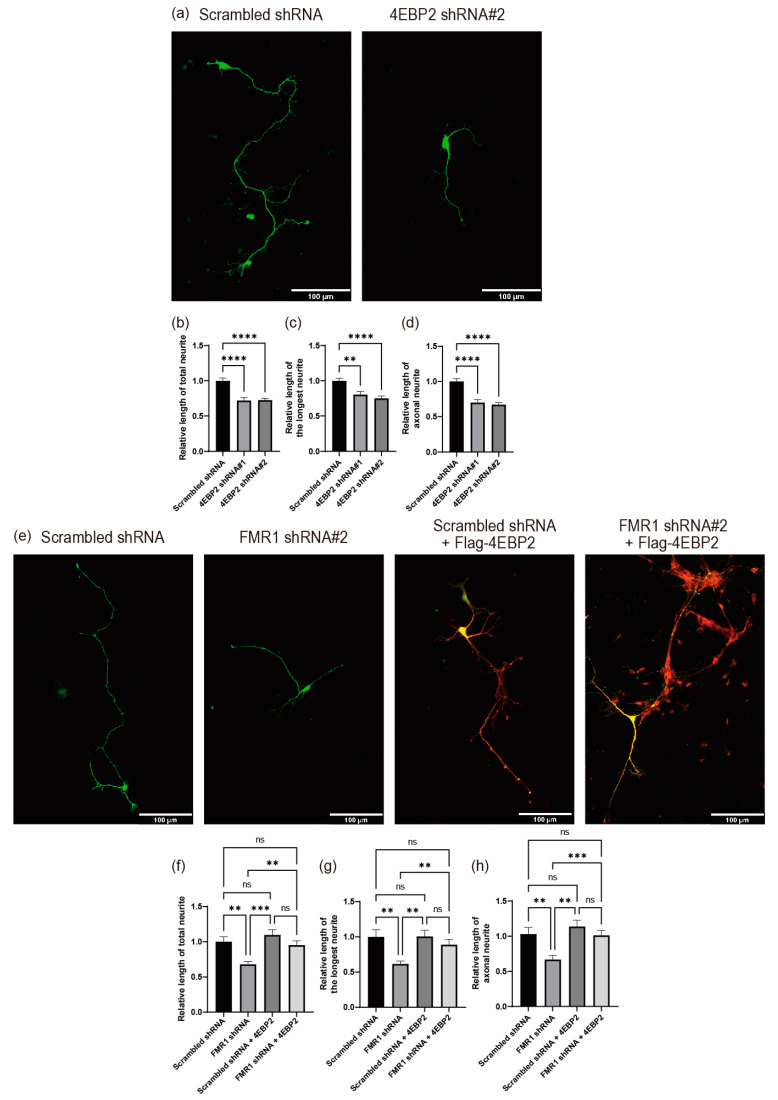
4EBP2 is epistatic to FMRP in the modulation of neurite outgrowth. (**a**–**d**) 4EBP2 appears to impact the normal process of neurite outgrowth in primary cultured hippocampal neurons. Plasmids expressing shRNA and the GFP encoding gene were transfected into cells after 24 h of neuronal cell culture, and the morphologies of neurites extending from the neuronal cells were analyzed at DIV 4 (Scrambled shRNA, *n* = 99 cells; *4EBP2* shRNA#1, *n* = 58 cells; *4EBP2* shRNA#2, *n* = 117 cells). (**a**) Representative pictures of the DNA-transfected neurons. The morphologies of neuronal cells expressing shRNAs (green) were analyzed and are depicted in the graphs (panels (**b**–**d**)). Utilizing the Simple neurite tracer plug-in of ImageJ (version 1.52p) program, the relative lengths of the total neurite (**b**), longest neurite (**c**), and axonal neurite (**d**) were calculated. Each value was normalized to the average neurite length of cells expressing negative control (Scrambled) shRNA. Two shRNAs (#1 and #2) with different core sequences were used. (**e**–**h**) Overexpression of 4EBP2 neutralized the effect of FMRP depletion on neurite outgrowth of primary cultured hippocampal neurons. A plasmid ectopically expressing Flag-tagged 4EBP2 (Flag-4EBP2) and another expressing shRNAs (Scrambled shRNA or *FMR1* shRNA#2) and GFP encoding gene were co-transfected into cells after 24 h of neuronal cell culture, and the morphologies of neurites extending from the neuronal cells were analyzed at DIV 4 (Scrambled shRNA, *n* = 35 cells; *FMR1* shRNA, *n* = 69 cells; Scrambled shRNA + 4EBP2, *n* = 21 cells; *FMR1* shRNA + 4EBP2, *n* = 62 cells). (**e**) Representative pictures of the DNA-transfected neurons. The expression of Flag-4EBP2 in cells was visualized by immunocytochemistry using an anti-Flag antibody conjugated with AlexaFluor568 showing red fluorescence (panels Scrambled shRNA + Flag-4EBP2 and *FMR1* shRNA#2 + Flag-4EBP2). The panels (Scrambled shRNA + Flag-4EBP2 and *FMR1* shRNA#2 + Flag-4EBP2) show a superimposed picture of the cells in green and red. Yellow areas indicate the presence of both GFP (green) and Flag-4EBP2 (red). The morphologies of neuronal cells expressing shRNA (green) and/or Flag-4EBP2 (red) were analyzed and are depicted in the graphs (panels (**f**–**h**)), as described in the legend of Figure 5. shRNA#2 against *FMR1* mRNA (Figure 5) was used for knockdown of *FMR1* mRNA. The scale bars in panel (**a**,**e**) represent 100 μm. Three or more independent repetitions were performed for each experiment. All data are presented as means ± SEM. In one-way ANOVA, the asterisks **, ***, and **** represent *p* < 0.01, *p* < 0.01, and *p* < 0.0001, respectively. *p* values over 0.05 are marked as non-significant (ns).

We investigated the role of 4EBP2 in neurite outgrowth, because the expression of 4EBP2 is modulated by FMRP. We used shRNAs targeting *4EBP2* mRNA (Supplementary Figure S5a) to test the effect of 4EBP2 knockdown on neurite outgrowth using a primary cell culture system of mouse hippocampal neurons. The depletion of 4EBP2 inhibited neurite outgrowth in mouse hippocampal neurons (Figure 6a–d). These results indicate that 4EBP2 has the potential to influence the regular process of neurite outgrowth.

We investigated whether the abnormal expression of 4EBP2, mediated by the reduction in FMRP, is the main mechanism of the FMRP defect-related pathological phenotype in neurite outgrowth because 4EBP2 appears to affect normal neurite outgrowth. We tested the effect of ectopic 4EBP2 expression against a background of FMRP knockdown (Figure 6e–h and Figure S5b). Ectopic expression of 4EBP2 did not affect neurite outgrowth in cells without FMRP depletion (Figure 6e–h). However, the ectopic expression of 4EBP2 almost completely restored neurite outgrowth in FMRP-depleted cells (Figure 6e–h). These results suggest that the pathological phenotype of neurite outgrowth caused by the FMRP defect is primarily attributable to reduced 4EBP2 expression mediated by the FMRP defect.

### 2.7. Inactivation of eIF4E Function Neutralizes the Defect in Neurite Outgrowth Caused by FMRP and/or 4EBP2 Deficiency

**Figure 7:**
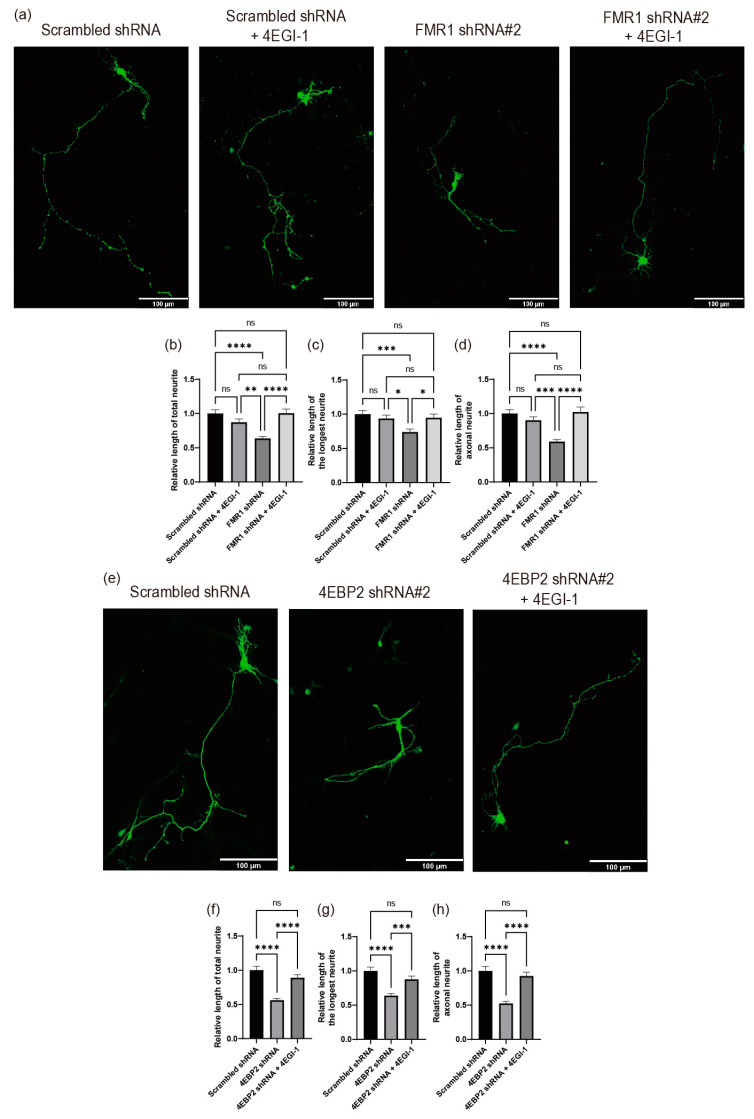
Inhibition of eIF4E activity neutralizes the effects of both FMRP- and 4EBP2-depletion on neurite outgrowth. (**a**–**d**) 4EGI-1 treatment neutralizes the effect of FMRP depletion on the neurite outgrowth of primary cultured hippocampal neurons. Plasmids expressing shRNA and GFP encoding gene were transfected into cells after 24 h of neuronal cell culture. Cells were treated with 50 μM of 4EGI-1 48 h after DNA transfection. The morphologies of neurites extending from the neuronal cells were analyzed at DIV 4 (Scrambled shRNA, *n* = 77 cells; Scrambled shRNA + 4EGI-1, *n* = 49 cells; *FMR1* shRNA, *n* = 83 cells; *FMR1* shRNA + 4EGI-1, *n* = 58 cells). (**a**) Representative pictures of the DNA-transfected neurons. The morphologies of neuronal cells expressing shRNAs (green) were analyzed and are depicted in the graphs (panels (**b**–**d**)), as described in the legend of Figure 5. The shRNA#2 against *FMR1* mRNA (Figure 5) was used in the experiments shown in panels (**a**–**d**). (**e**–**h**) 4EGI-1 treatment neutralizes the effect of 4EBP2 depletion on the neurite outgrowth of primary cultured hippocampal neurons. Plasmids expressing shRNA and GFP encoding gene were transfected into cells after 24 h of neuronal cell culture. Cells were treated with 50 μM of 4EGI-1 48 h after DNA transfection. The morphologies of neurites extending from the neuronal cells were analyzed at DIV 4 (Scrambled shRNA, *n* = 57 cells; *4EBP2* shRNA, *n* = 64 cells; *4EBP2* shRNA + 4EGI-1, *n* = 49 cells). (**e**) Representative pictures of the DNA-transfected neurons. The morphologies of neuronal cells expressing shRNAs (green) were analyzed and are depicted in the graphs (panels (**f**–**h**)), as described in the legend of Figure 5. The shRNA#2 against *4EBP2* mRNA (Figure 6) was used in the experiments shown in panels (**e**–**h**). The scale bars in panel (**a**,**e**) represent 100 μm. Three or more independent repetitions were performed for each experiment. All data are presented as means ± SEM. In one-way ANOVA, the asterisks *, **, ***, and **** represent *p* < 0.05, *p* < 0.01, *p* < 0.001, and *p* < 0.0001, respectively. *p* values over 0.05 are marked as non-significant (ns).

4EBP2 is a physiological inhibitor of eIF4E in neuronal cells [20,31,32,33]. The ASD-like phenotypes in FMRP-defective mice were relieved by treatment with 4EGI-1, a chemical inhibitor of eIF4E function [26,27]. We investigated the effects of 4EGI-1 on neurite outgrowth following the knockdown of either FMRP or 4EBP2 (Figure 7). As shown in Figure 5, the knockdown of *FMR1* mRNA reduced neurite outgrowth, which is consistent with previous reports [37,39,40,41,42]. Importantly, the defect in neurite outgrowth in the FMRP-depleted cells was effectively restored by the treatment with 4EGI-1 (Figure 7a–d). This confirms a previous report suggesting that pharmacological inhibition of the hyperactivated eIF4E, which is induced by FMRP defects, has the potential to ameliorate the FXS-like phenotypes [25].

Knockdown of *4EBP2* mRNA also hampered neurite outgrowth in neuronal cells (Figure 6). Importantly, the defect in neurite outgrowth in the 4EBP2-depleted cells was entirely reversed by the treatment with 4EGI-1 (Figure 7e–h). These results strongly suggest that 4EBP2 is involved in the biochemical pathways of FMRP loss-driven neurodevelopmental disorders.

## 3. Discussion

To understand the molecular basis of FXS caused by the abnormal expression of FMRP, we attempted to reveal the function of FMRP in modulating the normal outgrowth of neurites during the differentiation of primary cultured mouse hippocampal neurons (Figure 5). We found that normal levels of FMRP were required for the optimal expression of 4EBP2 in human neuroblastoma cells (SH-SY5Y), which differentiated into neuron-like cells following treatment with RA [43,44] (Figure 1). The modulation of 4EBP2 by FMRP occurred at the post-transcriptional level (Figure 1). Specifically, FMRP is required for the enhanced translation of 4EBP2, directed by the 5′UTR of *4EBP2* mRNA (Figure 2) where FMRP binds (Figure 3). This suggests that FMRP enhances the translation of *4EBP2* mRNA by interacting with the 5′UTR of *4EBP2* mRNA. We also found that an optimal amount of 4EBP2 is required for the normal outgrowth of neurites during the differentiation of hippocampal neurons (Figure 6a–d). Defective neurite outgrowth mediated by FMRP depletion was neutralized by the ectopic expression of 4EBP2 (Figure 6e–h), which suggests that the reduction in 4EBP2 in FMPR-depleted cells is the main cause of abnormal neurite outgrowth caused by a deficiency in FMRP.

Many reports have suggested that FMRP enhances the translation of specific mRNAs under specific conditions [12]. For example, the expression of Sod1 [14], Kv4.2 [15], and Dgkκ [16] was reduced in *FMR1* null mice, and human NOS1 translation was activated by FMRP in the developing neocortex [18]. Here, we report that FMRP enhances translation of *4EBP2* mRNA by binding to the 5′UTR of the mRNA under neuronal differentiation conditions. In an effort to reveal the mechanisms of translational enhancement of *4EBP2* mRNA by FMRP, we investigated whether the *4EBP2* 5′UTR has an internal ribosome entry site (IRES) function [61]. Hence, a plasmid producing an artificial di-cistronic mRNA containing two reporter genes and the *4EBP2* 5′UTR at the intergenic region was constructed, and reporter expressions were tested in SH-SY5Y cells. The *4EBP2* 5′UTR did not enhance the translation of the second gene under neuronal differentiation conditions (Supplementary Figure S2g). This result indicates that the *4EBP2* 5′UTR does not function as an IRES element. Therefore, FMRP does not function as an IRES trans-acting factor (ITAF) [62] for the translation of *4EBP2* mRNA.

In an effort to understand the mechanism of translational activator function of FMRP, we tried to find a translation factor that potentially recruits the 40S ribosomal subunit to *4EBP2* mRNA in concert with FMRP. For this purpose, we investigated whether DAP5 (an eIF4G1 homologue) participates in the FMRP-dependent translational activation during neuronal differentiation since DAP5 physically interacts with FMRP [56,60] and eIF4G1 homologues are associated with the 40S ribosomal subunit through interactions with eIF3 [54,55,56,57] (Figure 4). We confirmed the interaction between FMRP and DAP5 by coprecipitation of the proteins (Figure 4d). In addition, we revealed that the amount of FMRP-DAP5 complex increases in an RNA-dependent manner during neuronal differentiation. Moreover, we showed for the first time that the knockdown of *DAP5* mRNA reduces the expression of 4EBP2 during neuronal differentiation, similar to the knockdown of *FMR1* mRNA (Figure 4a–c). This result indicates that DAP5 also participates in the translational enhancement of *4EBP2* mRNA during neuronal differentiation. With these results, we can conjecture that FMRP enhances translation of *4EBP2* mRNA by forming a ternary complex composed of *4EBP2* mRNA-FMRP-DAP5 via RNA-protein–protein interactions during neuronal differentiation, and then DAP5 recruits the 40S ribosomal subunit to *4EBP2* mRNA through the interaction with eIF3.

DAP5 contains eIF3- and eIF4A-binding domains where the 40S ribosomal subunit associates and is known to mediate IRES-dependent translation [57]. However, the 5′UTR of *4EBP2* mRNA does not have IRES activity as described above. This indicates that DAP5 employs a different mechanism to facilitate the translation of *4EBP2* mRNA. Recent reports have shown that DAP5 mediates cap-dependent translation by interacting with eIF3d, which binds to the cap structure of certain mRNAs for translation [56,63]. Moreover, a recent report suggested that DAP5 facilitates translational re-initiation when mRNAs contain translationally active upstream open reading frames (uORFs) and G-quadruplex (GQ) in the 5′UTRs [64]. In this respect, it is noteworthy that the 5′UTR of *4EBP2* mRNA contains at least two translationally active uORFs starting from CUG codons, which were identified by ribosome profiling experiments using harringtonine [65,66] (Supplementary Figure S6). 

One of the remaining questions related to the FMRP- and DAP-mediated translation of *4EBP2* mRNA is how the 40S ribosomal subunit that is recruited to *4EBP2* mRNA recognizes the initiation codon of the main ORF for the translation of 4EBP2. One possible scenario is through a putative translational re-initiation by the 40S ribosome migrating to the mRNA by scanning from the 5′-end [64]. Alternatively, the 40S ribosome recruited to the mRNA by FMRP and/or DAP5 recognizes the main initiation codon by RNA looping of the intervening region between the 40S ribosome recruitment site and the initiation codon [67]. The latter hypothesis can explain the enigmatic mechanism of translational enhancement of TNF-α mRNA by the FXR1, an autosomal paralog of FMRP, which is also bound to the 3′UTR of the mRNA [68]. Despite these manifestations suggesting the involvement of FMRP and DAP5 in the translation of 4EBP2, further studies are required to understand the detailed mechanisms of translational enhancement of *4EBP2* mRNA by FMRP and/or DAP5.

In this study, we used human *4EBP2* mRNA and the SH-SY5Y cell line, a human neuronal model cell, in biochemical experiments to identify cellular proteins binding to the 5′UTR of *4EBP2* mRNA as well as the function of the 5′UTR in translational enhancement during neuronal differentiation. Based on our findings, we speculate that similar translational regulation of *4EBP2* mRNA would occur for many, if not all, mammalian *4EBP2* mRNAs, as the 5′UTRs of human and mouse *4EBP2* mRNAs have highly conserved primary sequences (71% of identity score) despite lacking evolutionary selection pressure of protein functions (Supplementary Figure S7). Moreover, they have conserved structural and functional features such as CUG-rich elements, putative G-quadruplex structures, and uORFs starting with CUG codons (the legend to Supplementary Figure S7). It is noteworthy that the translational initiation of uORF1 and uORF2 in human *4EBP2* mRNA has been experimentally proven [65] (Supplementary Figure S6) and that uORF1 and/or uORF2 of mouse *4EBP2* mRNA are actively translated [69]. The conserved sequences and motifs in the 5′UTRs of human and mouse *4EBP2* mRNAs strongly suggest that these two mRNAs potentially share the same regulatory mechanism at the post-transcriptional level. 

Moreover, the defect in neurite outgrowth caused by knockdown of either *FMR1* or *4EBP2* mRNA was neutralized by chemical treatment with 4EGI-1 (Figure 7), which inhibits the activity of eIF4E [26]. This indicates that the main function of FMRP and 4EBP2 in the normal outgrowth of neurites, which occurs in the early stages of neuronal development, is to inhibit eIF4E activity. This study focused on the early stages of neuronal development because we observed primary cultured hippocampal samples at 4 days after in vitro cultivation (DIV). This period is characterized by active axonal growth; however, it is too early to observe dendritic or spine growth. Moreover, SH-SY5Y cells are not a model system for testing FXS. Therefore, whether FMRP-dependent 4EBP2 expression also affects the later stages of neuronal development when dendritic spines are formed remains unclear. Nevertheless, we can speculate that the function of FMRP through enhanced 4EBP2 expression continues until the later stages of neuronal development, as the abnormal morphology and density of dendritic spines in FMRP-deficient cells were also neutralized by treatment with 4EGI-1 [27]. Notably, impaired synaptic function has been observed in 4EBP2 knockout mice [30,31], demonstrating the pivotal role of 4EBP2 in the development of neuronal cells.

## 4. Materials and Methods

### 4.1. Construction of Plasmids 

To construct plasmids expressing reporter genes containing full-length *4EBP2* 5′UTR or *4EBP2* 3′UTR, *4EBP2* 5′UTR and 3′UTR were obtained by PCR amplification from a human brain cDNA library (Panbionet, Pohang, Republic of Korea). The primers used in the PCR were as follows: full-length *4EBP2* 5′UTR was inserted into the 5′UTR region of the mono-cistronic reporter (forward: 5′-CCCCCGCTAGCGACCAGCTTCCCCAACTC-3′, and reverse: 5′-CCCCCGGCGCCGGGCCTTTCTTTATGTTTTTGGCGTCTTCGCGGCTCTGGCTGGGCTGGTGGCCGCTGCCGGCTGACGAGGACATGGCTGTGGGCGCG-3′), full-length *4EBP2* 5′UTR was inserted into the 5′UTR of the di-cistronic reporter (forward: the same sequence as full-length for mono-cistronic, and reverse: 5′-CCCCCTTCGAAGTGCGGCTCTGGCTGGGCTGGTGGCCGCTGCCGGCTGACGAGGACATGGCTGTGGGCGCG-3′), and *4EBP2* 3′UTR was inserted into the 3′UTR of the mono-cistronic reporter (forward: 5′-CCCGGTACCCTCTCCTGCAAGGATTAG-3′, and reverse: 5′-CCCGCGGCCGCGCAAGAATGGATATTTTGTGC-3′). The amplified DNA was ligated into the pcDNA3.1-*firefly* and p*Renilla*-cricket paralysis virus (CrPV) internal ribosome entry site (IRES)-*firefly*, respectively. 

To make fragmented *4EBP2* 5′UTR for the RNA pull-down assay, fragmented *4EBP2* 5′UTRs were obtained by PCR amplification from reporter plasmid containing full-length *4EBP2* 5′UTR. The primers used in the PCR were as follows: *4EBP2* 5′UTR for 1–186 (forward: the same sequence as full-length for mono-cistronic, and reverse: 5′-CCCCCCGGTACCTCGCTTCCTCCCGTTC-3′), *4EBP2* 5′UTR for 187–375 (forward: 5′-CCCCCGCTAGCGCGAGGAGCGCGCAG-3′, and reverse: 5′-CCCCCCGGTACCCGCCGGGCCTTTCTTTATG-3′), *4EBP2* 5′UTR for 1–236 (forward: the same sequence as full-length for mono-cistronic, and reverse: 5′-CCCCCC GGTACCTGCTTCGGCTCCTCAG-3′), *4EBP2* 5′UTR for 237–375 (forward: 5′-CCCCGCTAGCGCCCCG GCCCCGC-3′, and reverse: the same sequence as *4EBP2* 5′UTR for 187–375), and *4EBP2* 5′UTR for 89–193 G-quadruplex (GQ) containing region (forward: 5′-CCCCCGCTAGCGGCTGCTGGCTGAGGC-3′, and reverse: 5′-CCCCCCGGTACCTCCTCGCTC GCTTCCTC-3′). The amplified DNA was ligated into pcDNA3.1-*firefly*. 

To construct a plasmid expressing the mouse 4EBP2 or human DAP5, the corresponding CDS regions were obtained by PCR amplification from a mouse brain cDNA library or a human brain cDNA library, respectively. The primers used in the PCR were as follows: mouse 4EBP2 CDS (forward: 5′-CCCCCGATATCTCCGCGTCGGCCGGT-3′, and reverse: 5′-CCCGCGGCCGCTCAGATGTCCATCTCAAACTG-3′) and human DAP5 CDS (forward: 5′-CCCGGTACCGTGGAGAGTGCGATTGC-3′, and reverse: 5′-CCCGCGGCCGCTTAGTCAGCTTCTTCCTC-3′). The amplified DNA was ligated into the pcDNA3.1-3 × Flag. As previously mentioned, all shRNA constructs were created by cloning the 19–21 nt core sequences along with the loop sequence TTCAAGAGA into the pLentiLo × 3.7 vector [70,71]. The core sequences of the mouse *FMR1* shRNA responsible for clones #1, #2, and #3 were TCAGTCTTTCGGAGTAAA, GCTAGAAGCTTTCTGGAA, and GGAAGTAGACCAGTTGCG, respectively. The core sequences of mouse *4EBP2* shRNA responsible for clones #1 and #2 were GGAGGAACACGAATCATT and GCCTTAATTGAAGACTCC, respectively. CTACCGTTGTATAGGTG was the core sequence of the scrambled control shRNA. Mono-cistronic *Renilla*, EMCV IRES, PITSLRE IRES, and β-globin 5′UTR sequences have been previously described [67,72].

### 4.2. Cell Culture and Differentiation

Hippocampal tissues from E15 ICR mouse embryos were isolated in Hank’s Balanced Salt Solution (Gibco, Thermo Fisher Scientific, Waltham, MA, USA) and then dissociated in 0.25% trypsin (Sigma-Aldrich, St. Louis, MO, USA) and 0.1% DNase I (Sigma-Aldrich, St. Louis, MO, USA) for 10 min at 37 °C to establish primary cultures of hippocampal neurons. In neurobasal media (Gibco, Thermo Fisher Scientific, Waltham, MA, USA) supplemented with 10 mM HEPES (pH 7.4) and 10% (*v*/*v*) horse serum, cells were resuspended (4.0 × 10^5^ cells/mL final cell concentration) and then plated on glass coverslips that had been coated with poly-D-lysine and laminin. The cell media was changed to neurobasal medium containing 1% (*v*/*v*) penicillin/streptomycin, 2% (*v*/*v*) B27 supplement (Gibco, Thermo Fisher Scientific, Waltham, MA, USA), and 2 mM glutamine 2 h after culture. Lipofectamine 2000 reagent (11668019, Thermo Fisher Scientific, Waltham, MA, USA) was used to transfect neural cells after 24 h of culture, and 2 h after transfection, the medium was changed to culture medium.

Human SH-SY5Y neuroblastoma cells and mouse NIH/3T3 fibroblast cells were grown in DMEM (Gibco, Thermo Fisher Scientific, Waltham, MA, USA), which was adjusted to include 10% (*v*/*v*) fetal bovine serum (FBS) (Peak Serum, Wellington, CO, USA) and 1% (*v*/*v*) penicillin/streptomycin. The cells were cultivated at 5% CO_2_ and 37 °C. SH-SY5Y cells were differentiated in a differentiation medium consisting of DMEM supplemented with 10 µM RA (R2625, Sigma-Aldrich, St. Louis, MO, USA) and 1% (*v*/*v*) penicillin/streptomycin. Differentiating cells were maintained at 5% CO_2_ and 37 °C, avoiding exposure to light sources. After 48 h of incubation, the cells were lysed for further experiments.

SH-SY5Y or NIH/3T3 cells were seeded onto the appropriate plates. The cells were transfected with siRNA (200 nM final concentration) or plasmid using Lipofectamine 3000 reagent (L3000015, Thermo Fisher Scientific, Waltham, MA, USA) or FugeneHD (E231A, Promega, Madison, WI, USA), respectively, following the instructions of the manufacturer. The cell culture medium was replaced with fresh medium containing 10% (*v*/*v*) FBS 4 h after transfection. Protein levels were monitored via Western blotting at 48 or 72 h after transfection. The Scrambled siRNA sequence [73] is 5′-UUCUCCGAACGUGUCACGUdTdT-3′; the *FMR1* siRNA sequence [74] is 5′-CAGCUUGCCUCGAGAUUUCdTdT-3′; and the *DAP5* siRNA sequence [59] is 5′-AAUGUGGGUGUAGAGUCUAAAdTdT-3′. 

### 4.3. Animals

Primary hippocampal neurons were cultivated from mice that were purchased from Hyochang Science in Daegu, Republic of Korea. All animal procedures were approved by the Institutional Animal Care and Use Committee (IACUC) of Pohang University of Science and Technology (POSTECH-2022-0085). Each experiment was carried out according to the established procedures.

### 4.4. Immunocytochemistry

Solutions containing 4% paraformaldehyde in PBS or 4% paraformaldehyde and 4% sucrose in PBS were used to fix the cells for 20 min, followed by three washes with PBS. A solution containing 0.5% Triton X-100 in PBS was used to permeabilize the cells for 5 min, and 5% goat serum in PBS or 4% bovine serum albumin in PBS was used to block the cells for 30 min. For protein staining, cells were treated with secondary antibodies diluted in the blocking solution for 1 h at room temperature after being treated with primary antibodies diluted in the blocking solution for 1 h at room temperature or overnight at 4 °C.

### 4.5. In Vitro Neurite Outgrowth Assay

Primary cultured hippocampal neurons were transfected after 24 h of culture. In the recovery assay using 4EGI-1, neuronal cells were treated with 4EGI-1 for 48 h after plasmid transfection. At 72 h following transfection, 4% (*w*/*v*) paraformaldehyde and 4% sucrose were used to fix the neuronal cells for 20 min in PBS. An FV3000 confocal laser scanning microscope (Olympus, Tokyo, Japan) with a 20× objective lens was used to capture cell pictures, which were then processed using ImageJ (Fiji) software (version 1.52p) (RRID:SCR_002285, National Institutes of Health, Bethesda, MD, USA) [75].

### 4.6. Western Blotting and Antibodies

Immunoprecipitation buffer (IP) (40 mM HEPES–KOH (pH 7.5), 150 mM KCl, 0.1% NP40, 1 mM EDTA, 10 mM NaF, 10 mM β-glycerophosphate, 2 mM Na_3_VO_4_, and 1 mM PMSF) was used to lyse the cells. Sodium dodecyl sulfate-polyacrylamide gel electrophoresis (SDS-PAGE) was used to separate the proteins by mass and then the proteins were transferred to a PVDF membrane (IPVH00010, Millipore, Burlington, MA, USA). Chemiluminescence detection was performed using the Lumi Pico Solution (DG-WP250, DoGenBio, Seoul, Republic of Korea) and SuperSignal West Femto Maximum Sensitivity Substrate (34096, Thermo Fisher Scientific, Waltham, MA, USA). LAS (Image Quant LAS4000, GE Healthcare, Piscataway, NJ, USA) was used to visualize the chemiluminescence signals. TBS-T (0.1%) was used for washing and to prepare the blocking solution.

The following primary antibodies were used for Western blotting: anti-FMRP (4317, CST, Danvers, MA, USA), anti-4EBP2 (2845, CST, Danvers, MA, USA), anti-β-Actin (0869100-CF, MP Bio, Santa Ana, CA, USA), anti-DAP5 (610742, BD Biosciences, Franklin Lakes, NJ, USA), anti-Flag (F7425, Millipore, Burlington, MA, USA), and anti-GAPDH (4699-9555, AbD Serotec, Raleigh, NC, USA). The following secondary antibodies were used for Western blotting: Goat horseradish peroxidase (HRP)-anti-mouse IgG F(ab′)2 (31436, Thermo Fisher Scientific, Waltham, MA, USA) and Donkey HRP-anti-rabbit IgG (NA934V, GE Healthcare, Piscataway, NJ, USA). The following primary antibody was used for the immunostaining experiments: anti-Flag mouse monoclonal antibody (F1804, Sigma-Aldrich, St. Louis, MO, USA). The following secondary antibodies were used for immunostaining experiments: AlexaFluor488 conjugated goat anti-mouse antibody (A-11001, Thermo Fisher Scientific, Waltham, MA, USA), and AlexaFluor568 conjugated goat anti-mouse antibody (A-11004, Thermo Fisher Scientific, Waltham, MA, USA). 

### 4.7. Luciferase Assay

Mono- or di-cistronic reporter plasmids were transfected into SH-SY5Y cells one day after siRNA transfection. At 4 h after transfection, the medium was replaced with differentiation medium containing RA, and the cells were further incubated for 48 h. After that, the cells were collected and treated with passive lysis buffer (E194A, Promega, Madison, WI, USA). *Renilla* and *firefly* luciferase activities were measured using a dual-luciferase kit (E1960, Promega, Madison, WI, USA) according to the instructions of the manufacturer.

### 4.8. Quantitative RT-PCR Analysis

TRI-Solution (TS200-001, Bio Science Technology, Daegu, Republic of Korea) was used to purify total RNAs in accordance with the instructions of the manufacturer. Quantitative RT-PCR (qRT-PCR) analyses were performed as described previously [72]. The relative expression among the groups was calculated using the 2^−ΔΔCt^ method. 

The primer sequences for 4EBP2 and GAPDH were as follows: GAPDH (forward: 5′-TGCACCACCAACTGCTTAG-3′, and reverse: 5′-GAGGCAGGGATGATGTTC-3′) [72], and 4EBP2 (forward: 5′-AGTTTCTGTTGGATCGTCGCA-3′, and reverse: 5′-ACTGCATGTTTCCTGTCGTGA-3′).

The primer sequences for reporters were as follows: *Firefly* (forward: 5′-GAGGTTCCATCTGCCAGGTA -3′, and reverse: 5′-CCGGTATCCAGATCCACAAC-3′) [72], and *Renilla* (forward: 5′-TGTGCCACATATTGAGCCAG-3′, and reverse: 5′-CCAAACAAGCACCCCAATCA-3′) [76].

The primer sequences for confirming SH-SY5Y differentiation by RA treatment [43] were as follows: MAP2 (forward: 5′-CATGGGTCACAGGGCACCTATTC-3′, and reverse: 5′-GGTGGAGAAGGAGGCAGATTAGCTG-3′), Synaptophysin (forward: 5′-ATTGTGCCAACAAGACCGAGAGT-3′, and reverse: 5′-CAGGAAGATGTAGGTGGCCAGAG-3′), Laminin (forward: 5′-GTTTAACGATCCCAAAGTTCTCAAGTCC-3′, and reverse: 5′-GCAGGCATTCACTGGCACTTTCC-3′), and HMBS (forward: 5′-TCGGGGAAACCTCAACACC-3′, and reverse: 5′-CCTGGCCCACAGCATACAT-3′).

### 4.9. In Vitro Transcription and RNA Pull-Down Experiments with Biotinylated RNAs 

Plasmid templates used to produce biotinylated RNAs, including the full-length *4EBP2* 5′UTR, fragmented *4EBP2* 5′UTR, EMCV IRES, PISTLRE IRES, and β-globin 5′UTR [67,72], were linearized using restriction enzymes. Linearized DNAs were used for in vitro transcription utilizing T7 RNA polymerase with 1 mM biotinylated UTP, as previously described [72]. Lysates of differentiated SH-SY5Y cells (1000 μg) and biotinylated RNAs (100 μM) were used in the RNA pull-down assay, as previously described [72]. 

### 4.10. Flag-Immunoprecipitation

For the Flag-immunoprecipitation of mammalian cell extracts, the Flag-antibody-conjugated beads (A2220, Millipore, Burlington, MA, USA) were washed once with IP buffer to prepare them for use. The SH-SY5Y cells were lysed in IP buffer. Whole cell extract (WCE) was mixed with RNase A (R6513, Sigma-Aldrich, St. Louis, MO, USA) (or an equivalent volume of IP buffer) and then incubated for 30 min at 25 °C. WCEs were first pre-cleared for 1 h at 4 °C using Protein-G Agarose resin (5015952001, Roche, Basel, Switzerland) and then incubated for 1 h at 4 °C with Flag-antibody-conjugated beads while being continuously rotated. The proteins attached to the beads were isolated, resolved through SDS-PAGE, and examined via Western blotting after the beads were washed five times in IP buffer. 

### 4.11. Statistical Analysis 

GraphPad Prism 9.4.1 was used to conduct the statistical analysis. Western blotting, qRT-PCR, and luciferase assay data are presented as means ± SD. The neurite outgrowth data are presented as means ± SEM. The analysis of the data’s statistical significance was based on a one- or two-way analysis of variance (ANOVA) followed by a Bonferroni post hoc test for comparisons among several groups.

## 5. Conclusions

We discovered that 4EBP2 plays a key role in neuronal development and that FMRP functions as a translational activator by binding to the 5′UTR of *4EBP2* mRNA, thereby enhancing the translation of *4EBP2* mRNA during neuronal development. The translational inhibitory activity of FMRP in neuronal cells is, at least in part, mediated by the enhanced expression of 4EBP2 directed by FMRP because 4EBP2 is the major translational inhibitory protein that inactivates eIF4E function in neuronal cells.

## Data Availability

All data are available in the main text or the Supplementary Data. Ribosome profiling data shown in Supplementary Figure S6 are available at https://gwips.ucc.ie (accessed on 9 November 2023).

## Supplementary Materials

The following supporting information can be downloaded at: https://www.mdpi.com/article/10.3390/ijms242216319/s1.
