# Supplementary material for: FMRP Enhances the Translation of 4EBP2 mRNA during Neuronal Differentiation
Source: Int J Mol Sci. 2023 Nov 14;24(22):16319. doi: 10.3390/ijms242216319 (PMC10671300; doi:10.3390/ijms242216319)
Supplement: Supplementary file 1 [file ijms-24-16319-s001.zip › ijms-2672571-supplementary.pdf]

International Journal of
Molecular Sciences

MDPI

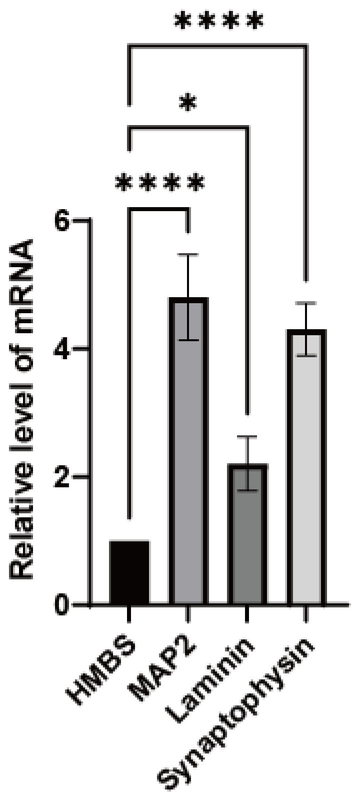

Supplementary Figure S1. Confirmation of SH-SY5Y cell differentiation

Relative amounts of mRNAs in neuronally differentiated SH-SY5Y cells compared to those in undifferentiated cells. The culture medium was replaced with fresh medium containing 10% (v/v) FBS or 10 μM RA at 48 h after SH-SY5Y cell culture. For purifying the mRNA, cells were lysed at 48 h after the media change. The amounts of mRNAs [one control (HMBS) and three neuronal markers (MAP2, Laminin, and Synaptophysin) mRNAs] in the cell lysates were measured using quantitative RT-PCR (qRT-PCR). The ratio of each mRNA (amount of mRNA in differentiated cells/amount of mRNA in undifferentiated cells) was normalized to that of HMBS. Three or more independent repetitions were performed for each experiment. All data are represented as means ± SD. In one-way ANOVA, the asterisks * and **** present $p<0.05$ and $p<0.0001$, respectively.

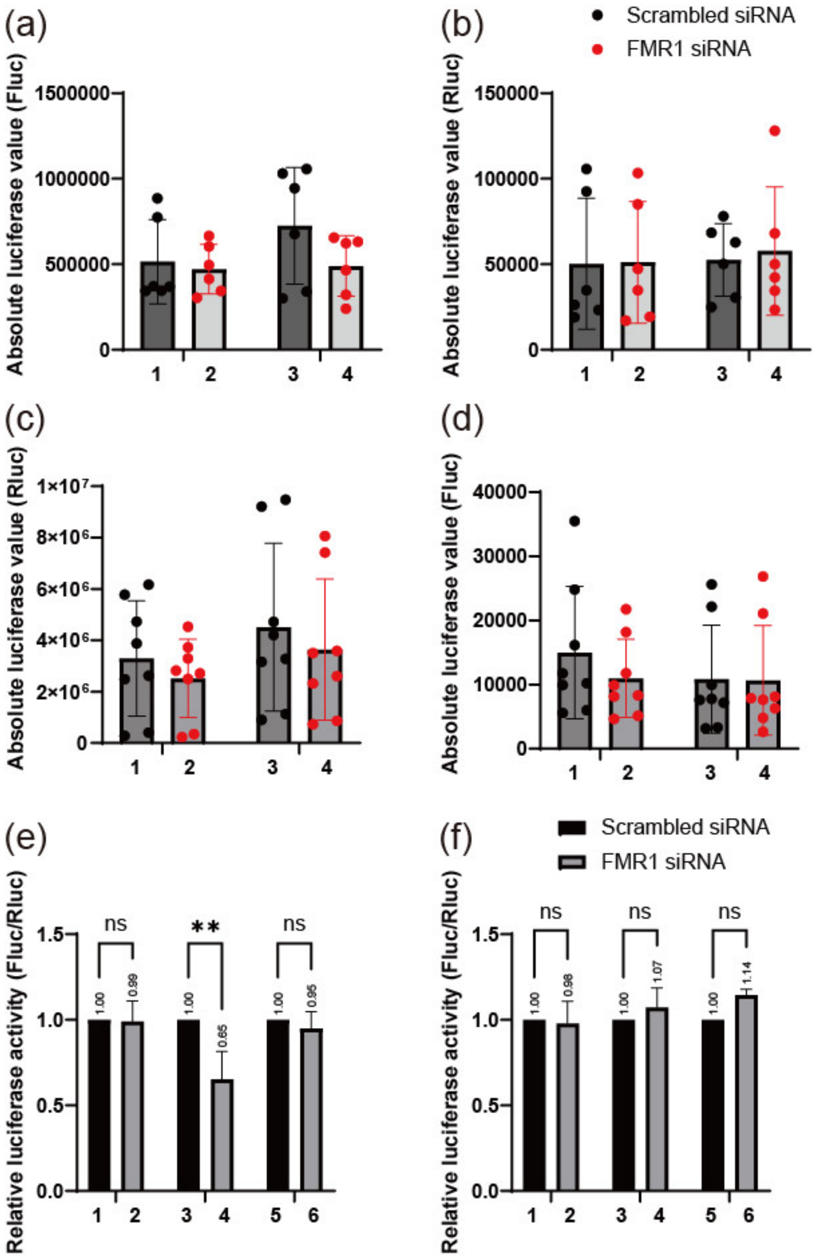

(g)

			Absolute luciferase value		Relative luciferase activity
			Fluc	Rluc	Fluc/Rluc
Rluc-EMCV IRES-Fluc	Scrambled siRNA	set1	726384	7433958	0.097712
		set2	387205	4513087	0.085796
	FMR1 siRNA	set1	609372	7034540	0.086626
		set2	414878	4307520	0.096315
Rluc-4EBP2 5'UTR-Fluc	Scrambled siRNA	set1	146	9895225	0.000015
		set2	148	6020578	0.000025
	FMR1 siRNA	set1	131	9918570	0.000013
		set2	155	6500100	0.000024

Supplementary Figure S2. The absolute values of luciferase activities

(**a-d**) Each dot represents the absolute value of luciferase activities. The luciferase activities in SH-SY5Y cells are depicted in lanes 1-2 (negative control mRNA) and lanes 3-4 (test mRNA) as described in Figure 2. (**a-b**) Luciferase activity data was obtained from the mono-cistronic mRNA system

shown in Figure 2. (**a**) The values of Fluc activities were used in Figures 2b and 2d. (**b**) The values of Rluc activities were used in Figures 2b and 2d. (**c-d**) Luciferase activity data was obtained from the di-cistronic mRNA system shown in Figure 2. (**c**) The values of Rluc activities were used in Figure 2f. (**d**) The values of Fluc activities were used in Figure 2f. (**e-f**) Luciferase activity data was obtained from the mono-cistronic mRNA system. SH-SY5Y cells were transfected with siRNAs (Scrambled siRNA for lanes 1, 3, and 5, and siRNA against *FMR1* mRNA for lanes 2, 4, and 6) after 24 h of cell culture. Plasmids expressing Fluc and Rluc mRNAs (negative control mRNA for lanes 1 and 2, test mRNA containing *4EBP2* 5′UTR for lanes 3 and 4, and test mRNA containing *4EBP2* 3′UTR for lanes 5 and 6) were transfected into cells 24 h after siRNA transfection. The culture medium was replaced with a fresh medium with (panel **e**) or without (panel **f**) neuronal differentiation 4 h after the DNA transfection. Following the medium change, cells were cultivated further for 48 h, and luciferase activities in each cell lysate were measured. The relative luciferase activities (Fluc/Rluc values) under various conditions are depicted with the Fluc/Rluc value of cell lysate treated with Scrambled siRNA set to ‘1’, as shown by the columns on lanes 1, 3, and 5. (**g**) Luciferase activities reflecting translation directed by encephalomyocarditis virus (EMCV) IRES or by *4EBP2* 5′UTR residing at the intergenic region of two reporter genes were analyzed. The translation of Fluc, which is directed by EMCV IRES, was used as a positive control IRES function. The transfection of SH-SY5Y cells and the analyses of reporter genes were performed as described in the legends to panel (**e**) except that the plasmids expressing di-cistronic mRNAs, instead of mono-cistronic mRNAs, were transfected into cells. The *4EBP2* 5′UTR did not induce translation of the second gene under neuronal differentiation conditions (see the absolute luciferase value in Fluc containing *4EBP2* 5′UTR), suggesting that the *4EBP2* 5′UTR does not function as an IRES element. Three or more independent repetitions were performed for each experiment. All data are represented as means ± SD. In two-way ANOVA, the asterisk ** indicates $p < 0.01$. p values over 0.05 are marked as non-significant (ns).

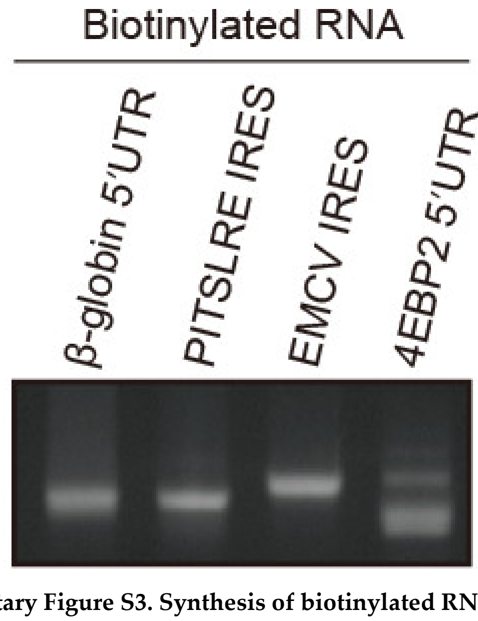

Supplementary Figure S3. Synthesis of biotinylated RNAs

T7 RNA polymerase was used for *in vitro* transcription to produce the indicated biotinylated RNAs. Multiple bands were observed in the lanes loaded with PITSLRE IRES and *4EBP2* 5'UTR RNAs. The multiple bands are likely to reflect the existence of multiple configurations of the RNAs.

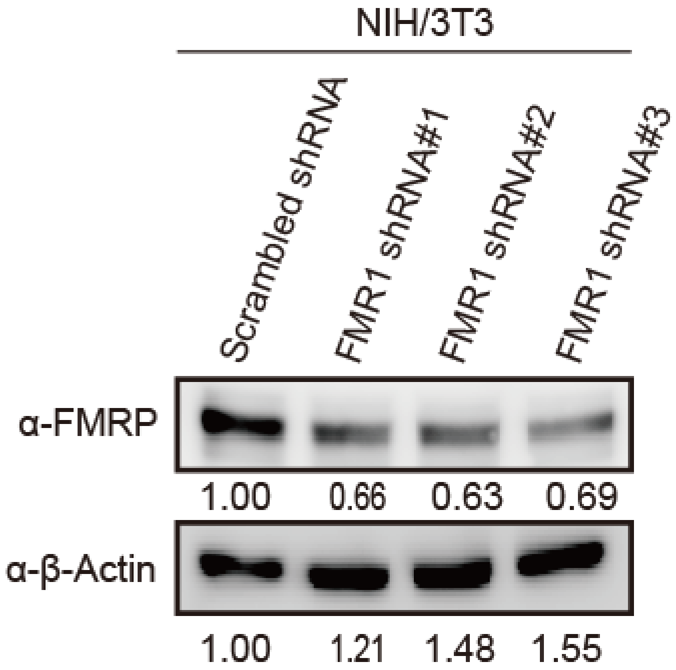

Supplementary Figure S4. Effects of DNAs expressing shRNAs against *FMR1* mRNA on FMRP levels in NIH/3T3 cells

The knockdown efficiencies of three different shRNAs were measured via western blotting. Plasmid was transfected into the cells 24 h after cell culture. The plasmid expressing shRNAs against *FMR1* mRNA also encodes GFP which is used for monitoring plasmid-transfected cells. Following DNA transfection, the cells were lysed 48 h later. The amounts of proteins (FMRP and β-Actin) in NIH/3T3 cells, transfected with plasmids expressing shRNAs#1, #2, and #3, were monitored via western blot analysis using the specified antibodies. From 31% to 37% of reduction of FMRP levels was observed from the pool of cells (panel α-FMRP). However, the reduction levels of FMRP were 46% to 58% when FMRP levels were normalized with the levels of β-Actin on each lane. This indicates that the real knockdown efficiencies of the *FMR1* shRNAs are very high (close to 100 %) considering that the transfection efficiency of the plasmids expressing both shRNAs and GFP into NIH/3T3 cells was about 50%, which was monitored by the expression of GFP in the plasmid-transfected cells with a fluorescence microscope. It is worth noting that the changes in the neurite outgrowth of primary cultured hippocampal neurons by knockdown of FMRP were monitored only for the GFP-expressing cells (Figures 5,6 and 7). Three or more independent repetitions were performed for each experiment.

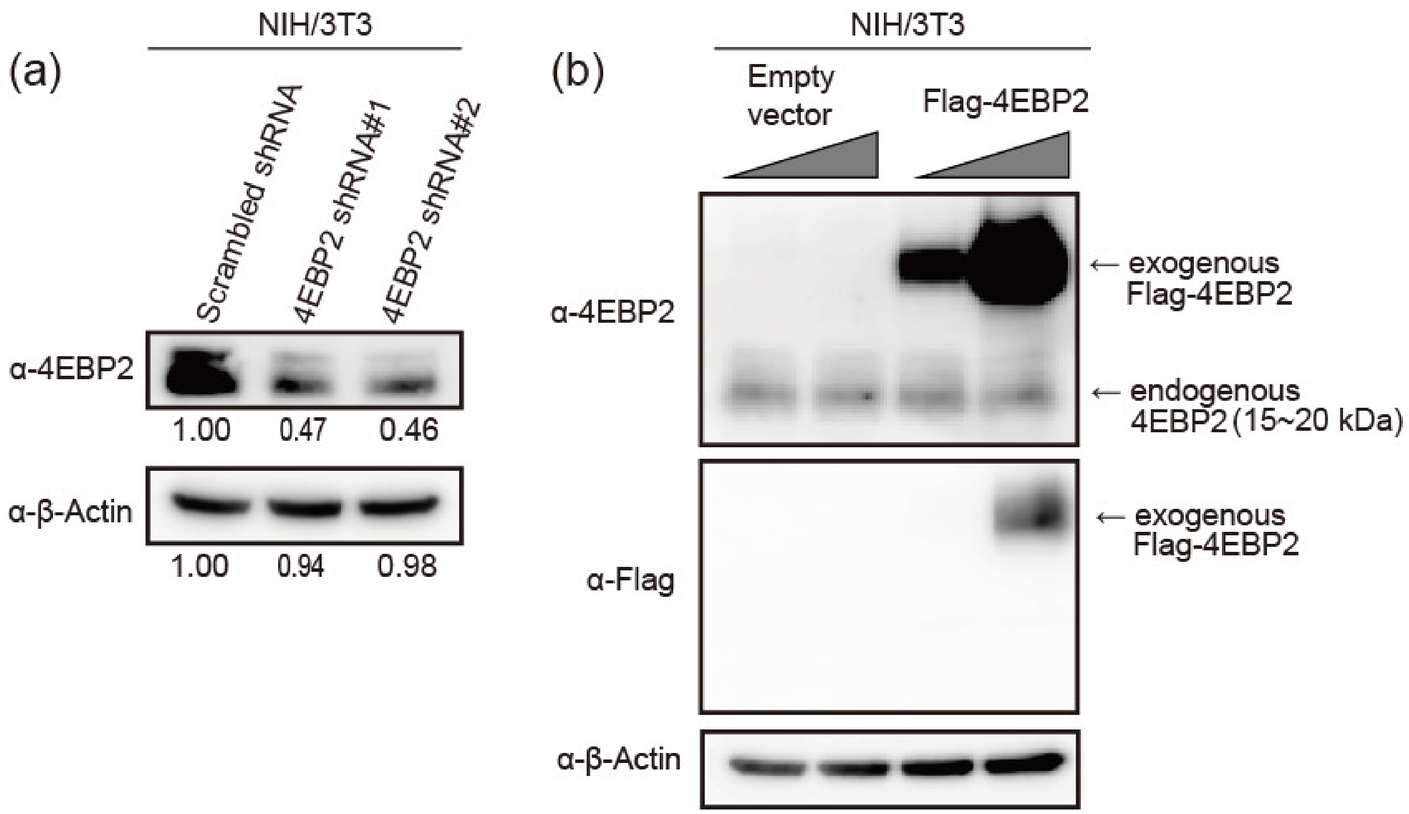

Supplementary Figure S5. Knockdown efficiencies of *4EBP2* mRNA by two shRNAs and ectopic expression of Flag-tagged 4EBP2

(**a**) The knockdown efficiencies of two different shRNAs were measured via western blotting. NIH/3T3 cells were transfected with plasmids expressing shRNAs#1 and #2 at 24 h after cell culture. The cells were then cultivated for a further 48 h before being lysed. The plasmid expressing shRNAs against *4EBP2* mRNA also encodes GFP which is used for monitoring plasmid-transfected cells. Following DNA transfection, the cells were lysed 48 h later. The amounts of proteins (4EBP2 and β-Actin) in NIH/3T3 cells, transfected with plasmids expressing shRNAs#1 and #2, were monitored via western blot analysis using the specified antibodies. From 53% to 54% of reduction of 4EBP2 levels was observed from the pool of plasmid-transfected cells (panel α-4EBP2). However, the reduction levels of 4EBP2 were 50% to 53% when FMRP levels were normalized with the levels of β-Actin on each lane. This indicates that the real knockdown efficiencies of the 4EBP2 shRNAs are very high (close to 100 %) considering that the transfection efficiency of the plasmids expressing both shRNAs and GFP into NIH/3T3 cells was about 50%, which was monitored by the expression of GFP in the plasmid-transfected cells with a fluorescence microscope. It is worth noting that the changes in the neurite outgrowth of primary cultured hippocampal neurons by knockdown of 4EBP2 were monitored only for the GFP-expressing cells (Figures 6 and 7). (**b**) Ectopic expression of Flag-tagged 4EBP2 (Flag-4EBP2) in NIH/3T3 cells. Plasmid expressing Flag-4EBP2 was transfected into NIH/3T3 cells 24 h after cell culture. The cells were then cultivated for a further 48 h before being lysed. The amounts of proteins (4EBP2, Flag-4EBP2, and β-Actin) in the cell lysates were monitored via western blot analysis using the specified antibodies. Three or more independent repetitions were performed for each experiment.

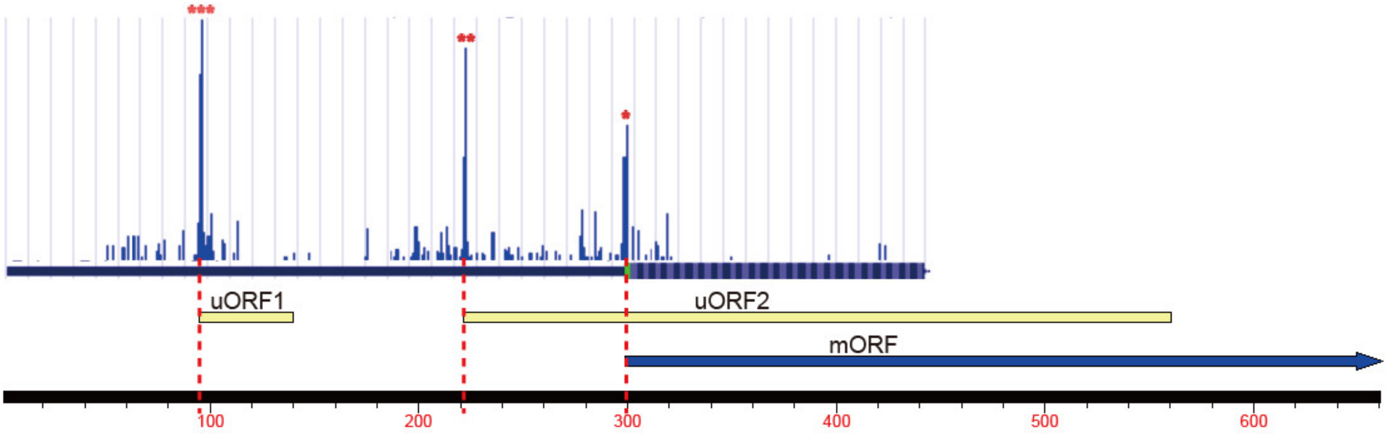

Human 4EBP2 mRNA
*, **, and ***: the 80S ribosome peaks
*: AUG codon
** and ***: CUG codons
uORF: upstream open reading frame
mORF: main open reading frame

Supplementary Figure S6. The ribosome profiling data on the 5'UTR of *4EBP2* mRNA

The CUG codons at the upstream open reading frames (uORF1 and uORF2) of human *4EBP2* mRNA are used as translation initiation codons. The ribosome profiling data on the 5'UTR of *4EBP*2 mRNA [65], which were acquired in the presence of harringtonine to arrest the 80S ribosome on the translation start sites [66], were accessed through the GWIPS-viz ribosome profiling database. The data indicate that both uORF1 and uORF2 are actively translated and the CUG codons at the beginning of the uORF1 and uORF2 are used as the start codons.

Human 4EBP2 mRNA
Length of 5′UTR: 297 bases
Length of uOFR1 (yellow): 45 bases
Length of uORF2 (yellow): 339 bases
uORF2 (yellow) vs. mORF (blue): out of frame
GQ (sky blue): a segment predicted to form G-quadruplex
CUG-rich element (purple): a segment containing repeated CUG sequence

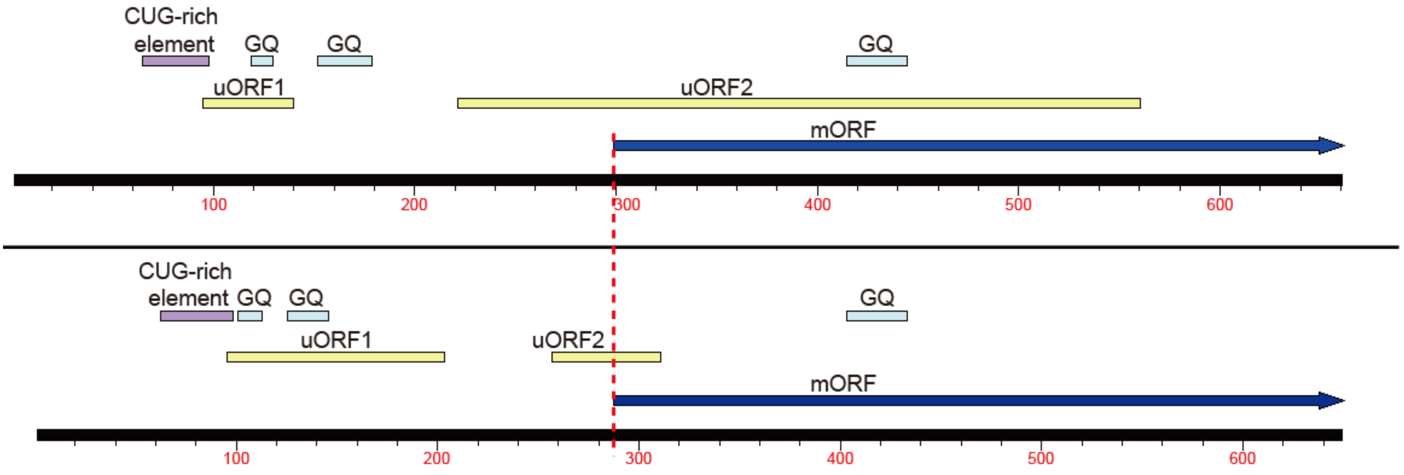

Mouse 4EBP2 mRNA
Length of 5′UTR: 286 bases
Length of uOFR1 (yellow): 108 bases
Length of uORF2 (yellow): 54 bases
uORF2 (yellow) vs. mORF (blue): out of frame
GQ (sky blue): a segment predicted to form G-quadruplex
CUG-rich element (purple): a segment containing repeated CUG sequence

Supplementary Figure S7. Similarities between the 5'UTRs of human and mouse *4EBP2* mRNAs

The 5'UTRs of human and mouse *4EBP2* mRNAs have very high level of primary sequence conservation (71% of identity score) even though they do not have evolutional selection pressure of protein functions. Moreover, they have conserved structural and functional features such as CUG-rich element composed of repeated CUG sequences, putative G-quadruplex (GQ) structures, and uORFs starting with CUG codons. In both human and mouse *4EBP2* 5'UTRs, the first uORF (uORF1) begins with a conserved CUG codon and ends after short coding sequences. In contrast, the second uORF (uORF2), which begins with a CUG codon, continues downstream of the main coding sequence of 4EBP2 (mORF). That is, the uORF2 and mORF overlap, but are out of frame for both human and mouse *4EBP2* mRNAs. Notably, translational initiation of uORF1 and uORF2 in human *4EBP2* mRNA has been experimentally demonstrated [65] (Supplementary Figure S6). Moreover, it has been reported that elongating ribosomes occupy the 5'UTR of mouse *4EBP2* mRNA [69], indicating that uORF1 and/or uORF2 of mouse *4EBP2* mRNA are actively translated. The conserved sequences and motifs in the 5'UTRs of human and mouse *4EBP2* mRNAs strongly suggest that these two mRNAs are most likely to share the same regulation mechanism at the post-transcriptional level.

References

65. Chen, J.; Brunner, A.D.; Cogan, J.Z.; Nuñez, J.K.; Fields, A.P.; Adamson, B.; Itzhak, D.N.; Li, J.Y.; Mann, M.; Leonetti, M.D.; et al. Pervasive functional translation of noncanonical human open reading frames. *Science* **2020**, *367*, 1140-1146, doi:10.1126/science.aay0262.
66. Ingolia, N.T.; Brar, G.A.; Rouskin, S.; McGeachy, A.M.; Weissman, J.S. The ribosome profiling strategy for monitoring translation in vivo by deep sequencing of ribosome-protected mRNA fragments. *Nat Protoc* **2012**, *7*, 1534-1550, doi:10.1038/nprot.2012.086.
69. Ingolia, N.T.; Brar, G.A.; Stern-Ginossar, N.; Harris, M.S.; Talhouarne, G.J.; Jackson, S.E.; Wills, M.R.; Weissman, J.S. Ribosome profiling reveals pervasive translation outside of annotated protein-coding genes. *Cell Rep* **2014**, *8*, 1365-1379, doi:10.1016/j.celrep.2014.07.045.
